# Crashworthiness of a Modular Assembled Multi-Cell CFRP Structure: Experimental and Numerical Investigation

**DOI:** 10.3390/ma19112405

**Published:** 2026-06-05

**Authors:** Tianli Chen, Hehe Kang, Huile Zhang, Pengpeng Zhi, Wei Wang, Zhonglai Wang

**Affiliations:** 1 Fuxi Laboratory, College of Life Science and Agronomy, Zhoukou Normal University, Zhoukou 466001, China; 2 School of Mechanical and Electrical Engineering, Zhoukou Normal University, Zhoukou 466001, China; 3 Yangtze Delta Region Institute (Huzhou), University of Electronic Science and Technology of China, Huzhou 313001, China; 4 School of Mechanical and Electrical Engineering, University of Electronic Science and Technology of China, Chengdu 611731, China

**Keywords:** bio-inspired structure, modular assembled multi-cell structure, carbon fiber reinforced polymer (CFRP), crashworthiness, TOPSIS

## Abstract

Lightweight thin-walled energy-absorbing structures play a critical role in passive safety systems for automotive and aerospace engineering applications, yet simultaneously achieving high specific energy absorption and stable crushing behavior remains a persistent challenge. Inspired by the topology of natural honeycombs, this study proposes a novel modular assembled multi-cell carbon fiber reinforced polymer (CFRP) structure (MAMCS), fabricated via a cost-effective modular assembly strategy based on a wrapping process. Quasi-static axial crushing experiments combined with validated finite element simulations were employed to systematically investigate the effects of inner layup configurations ([0°/90°], [30°/−60°], [45°/−45°]), cell number, and inner sub-cell size on crushing behavior. Among the investigated layup configurations, the [0°/90°] inner layup exhibited superior mean crushing force (*MCF*) and specific energy absorption (*SEA*). Multi-cell architectures significantly enhanced load-bearing capacity and crushing stability through mechanical interactions among internal sub-cells. Parametric analyses further revealed that enlarging the inner sub-cell size elevates both *MCF* and *SEA*, although at the expense of a higher peak crushing force (*PCF*). A TOPSIS-based multi-criteria decision-making framework was applied to identify a preferred configuration that achieves a favorable balance between peak load mitigation and energy absorption efficiency. The proposed MAMCS, characterized by its simple modular assembly, cost-effective fabrication, and superior crashworthiness performance, offers a promising bio-inspired design strategy for developing high-performance lightweight energy-absorbing structures in axial impact applications.

## 1. Introduction

Lightweight structures with high energy absorption are fundamental in automotive and aerospace applications, where passive safety and weight reduction represent simultaneous yet competing engineering objectives [1,2,3,4,5]. Thin-walled structures are widely employed as primary energy-absorbing components owing to their high specific energy absorption and ease of manufacture [6,7,8,9]. Among various strategies for improving crashworthiness, two approaches have emerged as particularly promising: multi-cell configuration design and the application of carbon fiber reinforced polymer (CFRP) materials [10,11,12]. Multi-cell architectures significantly enhance structural stability and energy absorption capacity through mechanical interactions among internal sub-cells [7,13], while CFRP materials offer exceptional specific strength, specific stiffness, and energy absorption efficiency [1,14,15,16,17,18]. These two strategies therefore provide a compelling foundation for the development of lightweight, high-performance crashworthy structures.

Multi-cell thin-walled structures have been extensively studied as an effective approach to improving crashworthiness. Compared with conventional single-cell tubes, multi-cell configurations generate additional corner elements and internal wall interactions under axial compression, which significantly enhance *MCF*, *SEA*, and crushing stability [6,19]. Drawing inspiration from biological architectures that have evolved over millions of years, researchers have further proposed a variety of bio-inspired multi-cell designs to exploit these interaction effects more efficiently. Chen et al. [7] introduced cactus-inspired bionic tubes and used quasi-static experiments and numerical simulations to demonstrate that the optimized cross-sectional configuration could increase *SEA* by up to 15.84% compared with the original design. Gao et al. [13] developed bio-inspired hierarchical multi-cell hexagonal tubes and found that increasing the hierarchical order progressively enhances *MCF* and *SEA*, with the third-order configuration achieving an *SEA* approximately 2.5 times that of the zeroth-order counterpart. Liu et al. [8] proposed 3D-printed multi-cell composite tubes and systematically investigated the effects of loading rate and temperature on crushing behavior, revealing that multi-cell configurations consistently outperformed single-cell tubes across a range of testing conditions. These studies collectively indicate that multi-cell configuration design, particularly when guided by bio-inspired principles, is a highly effective strategy for enhancing the crashworthiness of thin-walled energy-absorbing structures. However, the energy absorption efficiency of metallic multi-cell structures is fundamentally constrained by the high density of metallic materials, which limits their potential in applications with stringent weight-reduction requirements.

To overcome the weight limitations of metallic multi-cell structures, CFRP has been incorporated into multi-cell configurations to form hybrid structures [14]. Huang et al. [10] conducted static and dynamic axial crushing tests on Al/CFRP hybrid tubes with both single-cell and multi-cell sections, confirming that CFRP reinforcement substantially improves *SEA* while maintaining stable progressive crushing. Yu et al. [20] proposed a novel multi-cell CFRP/AA6061 hybrid tube and performed multi-objective structural optimization, demonstrating that the hybrid configuration achieves superior crashworthiness compared with its metallic counterparts. Ying et al. [21] experimentally investigated a bio-inspired second-order Al/CFRP hierarchical tube in which small-diameter CFRP sub-tubes were packed inside a large aluminum outer tube, and reported that the configuration with seven sub-structures achieved a 157.5% increase in *SEA* over the hollow aluminum baseline. More recently, research on hybrid multi-cell structures has been further extended to variable-thickness designs [22], diverse cross-sectional geometries [23], and refined finite element modeling approaches [24], collectively establishing Al/CFRP hybrid multi-cell structures as a well-studied and effective crashworthy solution. Nevertheless, these hybrid structures remain inherently dependent on metallic components: the metal constituents increase overall structural weight and density, the mismatch in thermal expansion coefficients between CFRP and metal raises long-term durability concerns, and the presence of metal fundamentally limits the achievable degree of lightweighting. These limitations naturally motivate the pursuit of CFRP multi-cell structures, which combine the superior material properties with the configuration advantages of multi-cell designs. However, studies on CFRP multi-cell structures remain scarce. Liu et al. [25] investigated the crushing responses of single-cell and double-cell CFRP square tubes and found that, contrary to the behavior observed in metallic structures, the double-cell CFRP tube exhibited lower *SEA* than its single-cell counterpart. This was attributed to insufficient deformation in the T-shaped junction regions, where the inner laminae splayed completely outward rather than crushing progressively, thereby reducing the energy absorption capacity. This finding highlights a fundamental challenge: the direct transfer of metallic multi-cell topologies to CFRP structures is ineffective, and the development of CFRP multi-cell structures requires both a rethinking of structural configurations and a viable manufacturing strategy. However, to the best of our knowledge, no existing study has proposed a fully CFRP multi-cell structure that simultaneously addresses this configurational challenge through a dedicated modular assembly strategy—one in which individual sub-cells are independently fabricated and subsequently integrated via adhesive bonding and outer-layer wrapping, without reliance on any metallic components. The key distinction of the proposed MAMCS from existing work is therefore threefold: (i) it achieves multi-cell interaction benefits in a fully metal-free CFRP system, overcoming the weight and durability limitations of Al/CFRP hybrid designs; (ii) the modular assembly strategy eliminates the problematic monolithic T-shaped junctions identified in prior pure CFRP multi-cell studies, enabling controlled progressive crushing at each sub-cell interface; and (iii) by independently controlling the layup of each sub-cell, the failure mode and energy absorption mechanism can be tailored in a way that is not possible with monolithically formed CFRP multi-cell tubes.

To address these challenges, this study proposes a novel modular assembled multi-cell CFRP structure (MAMCS) inspired by the topology of natural honeycombs. By adopting a modular assembly strategy, fabricating individual CFRP sub-cells independently and subsequently bonding them into a multi-cell configuration with an outer wrapping layer, the proposed approach effectively bypasses the manufacturing constraints associated with conventional CFRP forming processes. This enables the realization of CFRP multi-cell structures in a cost-effective and reproducible manner. Quasi-static axial crushing experiments, combined with high-fidelity finite element simulations, were conducted to systematically investigate the effects of inner layup configurations, cell number, and inner sub-cell size on crashworthiness. The results demonstrate that the [0°/90°] inner layup and multi-cell architecture provide superior energy absorption performance, and that increasing the inner sub-cell size ratio elevates both *MCF* and *SEA* at the cost of a higher *PCF*. A TOPSIS-based multi-criteria decision-making framework was employed to identify the preferred configuration that balances peak load mitigation and energy absorption efficiency. This work confirms that modular assembly is a practical strategy for developing high-performance CFRP multi-cell energy-absorbing structures, offering useful guidance for lightweight crashworthy design in automotive and aerospace applications. The remainder of this paper is organized as follows: Section 2 describes the structural design and fabrication of the MAMCS; Section 3 presents the quasi-static crushing experiments; Section 4 details the finite element modeling, parametric analysis, and optimal configuration selection; and Section 5 summarizes the main conclusions.

## 2. Design and Fabrication of a Novel Modular Assembled Multi-Cell CFRP Structure (MAMCS)

**Figure 1:**
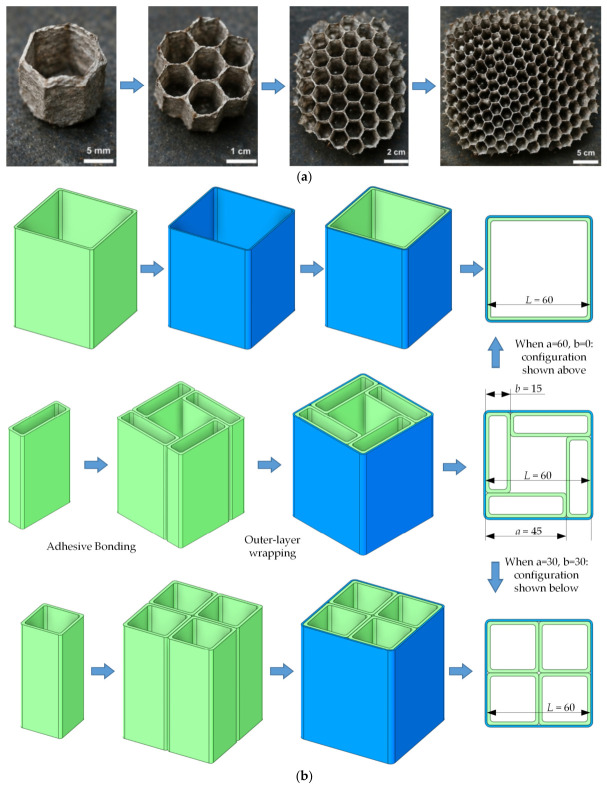
Schematic of the novel modular assembled multi-cell CFRP structure (MAMCS) inspired by natural honeycomb: (**a**) Natural honeycomb pattern; (**b**) Corresponding MAMCS design.

Inspired by the topology of natural honeycomb structures, this study proposes a novel modular assembled multi-cell CFRP structure (MAMCS), as shown in Figure 1. The MAMCS was fabricated using a modular assembly strategy. First, thin-walled inner sub-cells were manufactured as basic structural units. These sub-cells were then arranged and adhesively bonded to form a multi-cell core. Finally, the assembled core was wrapped with CFRP layers on its outer surface to complete the fabrication of the MAMCS. Detailed descriptions of the structural design and fabrication process are provided in the subsequent sections.

### 2.1. Structural Design of the MAMCS

The configuration of the MAMCS can be adjusted by changing the dimensions of the inner thin-walled sub-cells, thereby generating a series of derived configurations. These configurations exhibit varying structural geometries and energy absorption characteristics. Considering manufacturability and experimental cost, square-section CFRP modular assembled multi-cell structures and their derived configurations were selected for investigation.

As shown in Figure 1b, the proposed structure consists of four inner sub-cells and an outer wrapping layer. The side lengths of the inner sub-cells are defined as *a* and *b*, while the inner side length of the outer wrapping layer is denoted as *L*. These dimensions satisfy the following geometric relationships: 0 < *a* < *L*, 0 < *b* < *L*, and *a* + *b* = *L*. By varying the parameter *a*, different structural configurations can be obtained. For example, when *a* = *L* (and *b* = 0), the structure corresponds to a single-cell configuration; when *a* = *b* = *L*/2, the structure becomes a four-cell configuration.

### 2.2. Fabrication Process of the MAMCS

**Figure 2:**
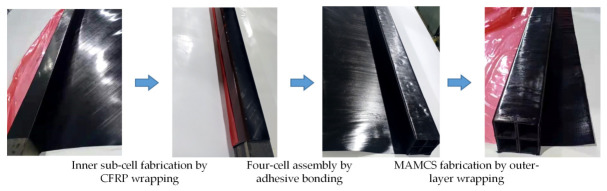
Schematic illustration of the fabrication process of the MAMCS.

In view of the lightweight design objective, T300/E51 prepreg (manufactured by Toray Industries, Inc., Tokyo, Japan) was selected for both the inner sub-cells and the outer wrapping layer. Common fabrication methods for CFRP thin-walled tubes include hand lay-up, filament winding [26], pultrusion, and resin transfer molding (RTM). Since the specimens in this study were fabricated in small batches and involved multiple layup angles, a wrapping process was adopted for both the inner sub-cells and the outer wrapping layer based on considerations of fabrication cost and process controllability [27]. The fabrication process is illustrated in Figure 2.

Different layup configurations have a significant influence on the crashworthiness of CFRP structures. In this study, 0° refers to the axial direction of the thin-walled tube and 90° refers to the circumferential direction. Drawing on established findings regarding layup effects on CFRP crashworthiness [24], three layup schemes were adopted for the inner sub-cells: [0°/90°], [30°/−60°], and [45°/−45°]. For the outer wrapping layer, a [90°] layup was used in all specimens to ensure circumferential stability under axial loading. The detailed layup configurations are summarized in Table 1. It is noted that the inner sub-cell stacking sequences ([0°/90°]_5_, [30°/−60°]_5_, and [45°/−45°]_5_) are not symmetric about the mid-plane. This results from the continuous wrapping fabrication process, in which achieving a symmetric laminate would substantially increase fabrication complexity. For thin-walled tubes under axial crushing, the effect of bending-stretching coupling introduced by laminate asymmetry is considered secondary compared to the dominant in-plane and interlaminar failure mechanisms, and is further mitigated by the circumferential constraint provided by the outer [90°]_6_ layer.

Specimen notation is defined as follows: C1 and C4 denote specimens with one and four inner sub-cells, respectively; N0, N30, and N45 represent the inner layups of [0°/90°]_5_, [30°/−60°]_5_, and [45°/−45°]_5_, respectively; W90 denotes the outer layup of [90°]_6_. For example, C4-N45-W90 corresponds to a four-cell modular assembled thin-walled structure with an inner layup of [45°/−45°]_5_ and an outer layup of [90°]_6_. The outer wrapping layer was fixed at [90°]_6_ across all specimens to provide circumferential constraint and ensure structural stability under axial loading, and to serve as a controlled variable in the parametric study. While alternative outer layup configurations may offer additional opportunities for performance improvement, their systematic investigation is beyond the scope of the present study and will be considered in future work.

The fabrication of the MAMCS mainly involved the following steps.

**Step 1: Fabrication of inner thin-walled sub-cells:** Toray T300-12K carbon fiber prepreg (T300/E51; Toray Industries, Inc., Tokyo, Japan) was used for the inner sub-cells. Square aluminum mandrels were first polished and cleaned with acetone to ensure smooth and impurity-free surfaces. Prior to wrapping, a silicone-based release agent was applied to the mandrel surfaces to facilitate demoulding. The CFRP prepreg was then wrapped onto the mandrel surface using the wrapping process according to the layup angles listed in Table 1 to form the inner single-cell structures. After wrapping, the specimens were sealed in vacuum bags and cured in an autoclave. The curing parameters were as follows: preheating at 80 °C for 35 min, followed by pressurization with a temperature increase to 135 °C for 100 min under a pressure of 0.4 MPa. Following the autoclave curing cycle, the CFRP tubes were removed from the mandrels by axial sliding assisted by light mechanical tapping. The slight differential thermal contraction between the CFRP tube and the aluminum mandrel upon cooling facilitated clean separation without surface damage.

**Step 2: Surface treatment and assembly of multi-cell core:** The cured CFRP single-cell tubes were sanded using a 45° cross-hatching pattern to increase surface roughness and improve bonding performance. For the four-cell configuration, four single-cell tubes were adhesively bonded using 3M DP460 two-part epoxy adhesive (3M Company, St. Paul, MN, USA). The assembled multi-cell core was allowed to cure at room temperature for 24 h to achieve full bond strength before subsequent processing.

**Step 3: Fabrication of outer wrapping layer:** The assembled multi-cell core served as a mandrel for the outer layer. T300/E51 prepreg was wrapped around the outer surface of the core at a 90° circumferential angle to ensure stability under axial loading. The assembly was then vacuum-sealed and cured in an autoclave using the same curing parameters as those used for the inner sub-cells.

**Step 4: Cleaning:** All specimens were cleaned with acetone to remove surface contaminants.

**Figure 3:**
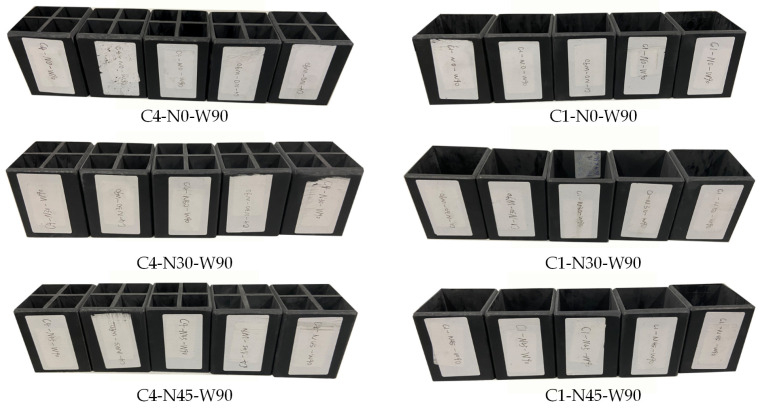
Fabricated MAMCS specimens with different layup configurations.

**Step 5: Final preparation:** All specimen configurations listed in Table 1 were fabricated following the above procedure. A 45° chamfer (trigger) was machined at one end of each specimen to initiate progressive crushing. The final specimens after chamfering are shown in Figure 3.

## 3. Quasi-Static Axial Crushing Experiments

Quasi-static axial crushing experiments were conducted on the MAMCS specimens to evaluate their energy absorption performance. Key crashworthiness indicators, including *MCF*, total energy absorption (*EA*), and *SEA*, were obtained from the tests. The experimental results were analyzed to investigate the crushing deformation modes and energy absorption characteristics of the proposed structure, thereby revealing the failure mechanisms and energy dissipation behaviors under different inner layup configurations and varying numbers of sub-cells.

### 3.1. Experimental Setup and Procedure

**Figure 4:**
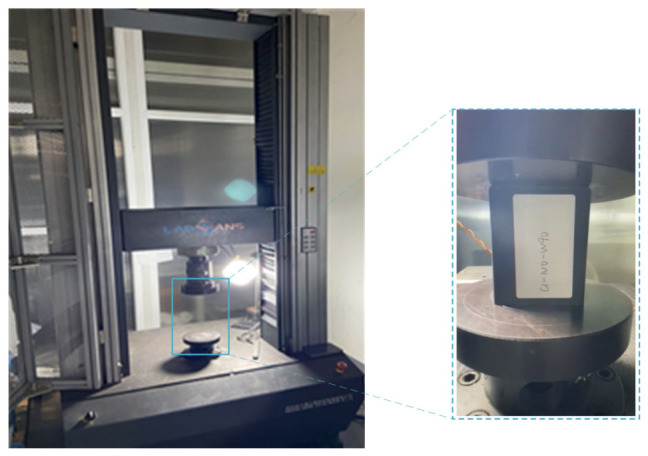
Schematic of the WDW-200 kN experimental setup.

Quasi-static axial crushing tests were conducted using a WDW-200 kN universal testing machine (Shenzhen SANS Testing Machine Co., Ltd., Shenzhen, China). The experimental setup is shown in Figure 4. A dedicated data acquisition system was used to record the crushing force, while an industrial camera was employed to capture the crushing process and final failure modes.

The experimental procedure was as follows. Each specimen was centrally positioned on the fixed lower platen of the testing machine. A preload was first applied at a rate of 1 mm/min until the force reached approximately 1 kN. This preload ensured that the specimen was securely seated and its axis was properly aligned with the loading direction. Axial crushing was then performed at a constant displacement rate of 5 mm/min, consistent with established quasi-static testing practice in the CFRP crashworthiness literature [16,17]. At this displacement rate, the nominal strain rate is approximately 1.39 × 10^−3^ s^−1^ (based on the specimen height of 60 mm), which falls well within the quasi-static regime, and strain-rate effects are therefore considered negligible for all tested configurations [8]. The test was terminated when the specimen was compressed by 30 mm, corresponding to 50% of the original specimen height. This termination criterion ensures that the complete stable crushing plateau is captured while avoiding the onset of densification. During the crushing process, the deformation behavior was recorded by the industrial camera at a fixed angle. Force–displacement curves were recorded throughout the crushing process for each specimen. After testing, the final crushed morphology of each specimen was documented photographically.

### 3.2. Crashworthiness Indicators

**Figure 5:**
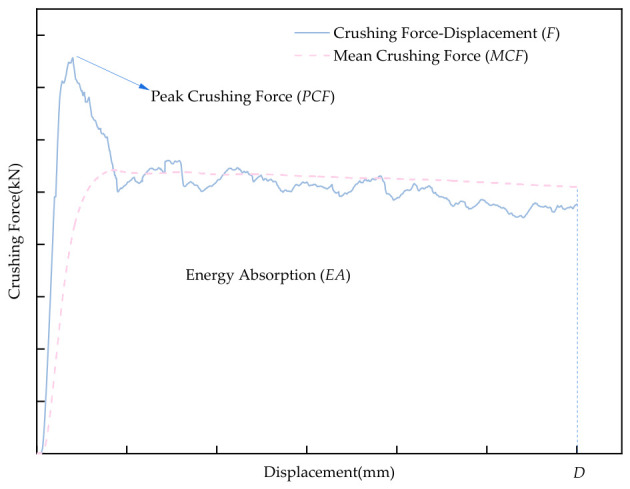
Typical crushing force–displacement curve and definitions of crashworthiness indicators.

To evaluate the crashworthiness of the proposed MAMCS specimens under quasi-static axial crushing, several widely used indicators were employed in this study, including peak crushing force (*PCF*), mean crushing force (*MCF*), energy absorption (*EA*), and specific energy absorption (*SEA*), as shown in Figure 5. These indicators are subsequently employed in a TOPSIS-based multi-criteria decision-making framework (Section 4.2.2) to identify the preferred structural configuration. In this framework, *PCF* is treated as a cost-type criterion (to be minimized) to limit the initial impact load transmitted to occupants or payload, while *MCF* and *SEA* are treated as benefit-type criteria (to be maximized) to reflect load-bearing capacity and energy absorption efficiency, respectively. *SEA* is assigned a higher weight (0.5) relative to *PCF* and *MCF* (0.25 each), reflecting its primacy as the most representative indicator of energy absorption efficiency for lightweight structures.

The peak crushing force (*PCF*) represents the maximum load recorded during the crushing process and reflects the initial impact resistance of the structure, as illustrated in Figure 5. Energy absorption (*EA*) is defined as the total energy absorbed by the structure during the crushing process and can be expressed as follows:

(1) $$EA=\int_{0}^{D}F\left(x\right)dx$$

where *F*(*x*) represents the crushing force–displacement curve, *x* denotes the crushing displacement, and *D* is the total crushing displacement, which corresponds to 50% of the original specimen height and lies entirely within the stable progressive crushing stage for all tested configurations.

Mean crushing force (*MCF*) is defined as the average load sustained during the stable crushing stage and can be calculated as follows:

(2) MCF=EAD

To account for the influence of structural mass, the specific energy absorption (*SEA*) is also defined as the energy absorbed per unit mass of the structure and can be expressed as follows:

(3) SEA=EAM

where *M* is the mass of the structure. Here, *M* denotes the total mass of the fully assembled MAMCS specimen, including the inner CFRP sub-cells, the adhesive bonding layer, and the outer [90°]_6_ wrapping layer, as measured by direct weighing of the complete specimen prior to testing.

### 3.3. Experimental Results and Discussion

#### 3.3.1. Effects of Layup Angle on Crashworthiness

**Figure 6:**
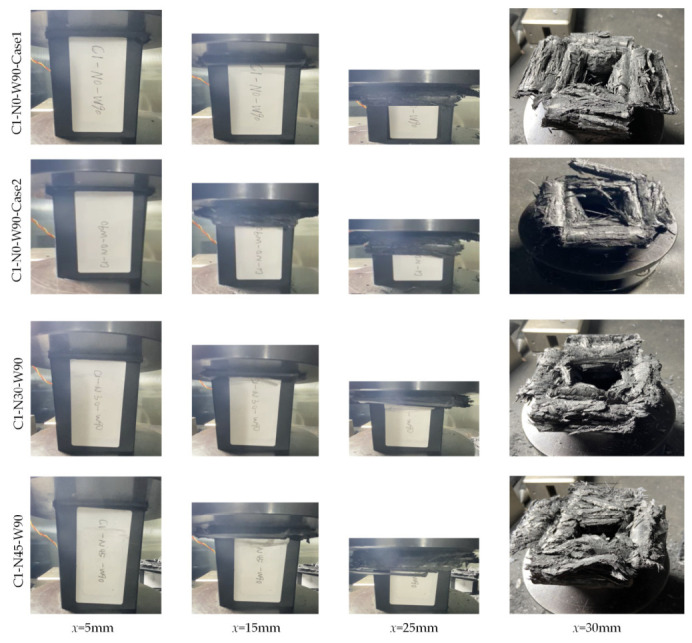
Effects of layup angle on deformation modes of single-cell MAMCS.

**Figure 7:**
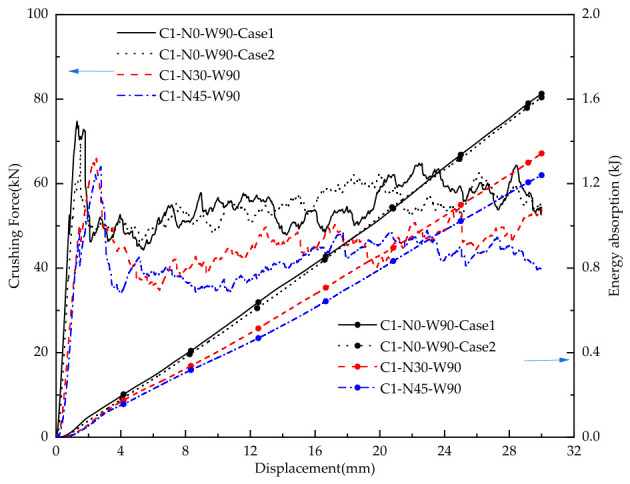
Effects of layup angle on the crushing force–displacement and *EA*–displacement responses of single-cell MAMCS.

Initially, two C1-N0-W90 cases were tested, and the results are shown in Figure 6 and Figure 7. As illustrated in Figure 6, both specimens exhibited progressive failure, initiating from the chamfered trigger at the top end. Typical failure modes, including corner cracking, fiber fracture, and delamination, were observed. Figure 7 shows that the force–displacement and energy absorption–displacement curves of the two specimens are in excellent agreement, indicating high repeatability of the experiments. The repeatability of the experimental setup was verified through duplicate tests on the C1-N0-W90 configuration, which showed excellent agreement between the two specimens (Figure 7). Based on this result and considering fabrication cost constraints, the remaining configurations were each tested once, consistent with established practice in the CFRP crashworthiness literature [16,17]. The quantitative comparisons reported in Section 3.3 should therefore be interpreted as indicative trends rather than statistically validated differences, and further replication will be pursued in future work.

Comparison of the crushing deformation modes under different inner layup configurations shows that the C1-N0-W90, C1-N30-W90, and C1-N45-W90 specimens all exhibited progressive failure, initiating from the chamfered trigger at the top end. Corner cracking was observed in all specimens. During the tests, cracks propagated in both the fibers and the matrix, often accompanied by audible cracking sounds. Crack propagation was followed by pronounced ply bending and brittle fracture.

Delamination caused the outer layers to fold outward and the inner layers to fold inward, resulting in a petal-like appearance with clearly defined boundaries. Fiber bundles in both the inner and outer layers experienced partial breakage during bending. As the crushing displacement increased, the bent region of the outer-layer fibers expanded, and some fiber bundles exhibited downward deflection. The inward-folded inner bundles, being confined within the tube, were fractured more severely than the outward-folded bundles and gradually filled the remaining interior cavity as crushing progressed.

**Figure 8:**
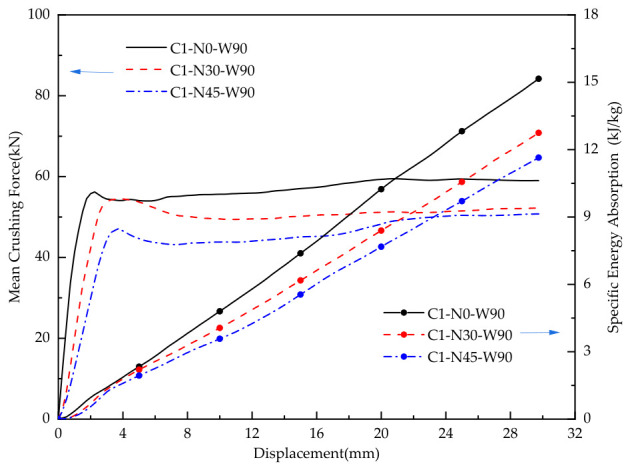
Effects of layup angle on the *MCF*–displacement and *SEA*–displacement responses of single-cell MAMCS.

Analysis of the force–displacement curves and energy absorption data for the specimens shown in Figure 7 and Figure 8 reveals consistent trends among the three inner layup configurations. All specimens exhibited progressive crushing. After reaching the peak force, the crushing force dropped sharply, followed by a stable crushing stage. Among the three layups, the C1-N0-W90 specimens reached the highest peak force and maintained higher loads during the stable phase.

The *MCF* of C1-N0-W90 was 12.8% and 16.4% higher than those of C1-N30-W90 and C1-N45-W90, respectively. Similarly, the total energy absorption (*EA*) and specific energy absorption (*SEA*) of C1-N0-W90 were 19.7% and 31.0% higher than those of C1-N30-W90 and C1-N45-W90, respectively. These results demonstrate that the [0°/90°] inner layup provides superior axial load-carrying capacity and enhanced energy absorption for single-cell MAMCS specimens.

**Figure 9:**
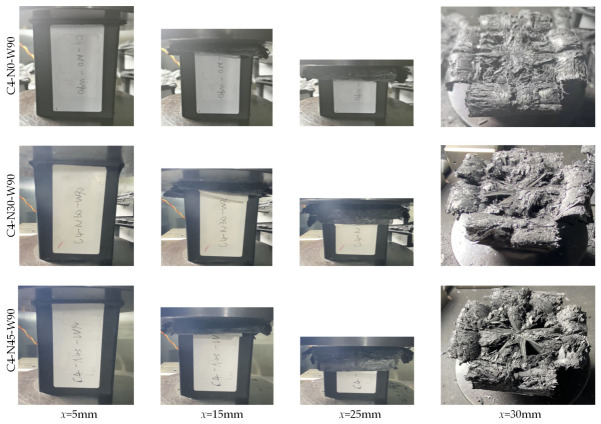
Effects of layup angle on deformation modes of four-cell MAMCS.

Figure 9 compares the crushing deformation modes of four-cell MAMCS specimens with different inner layup configurations. The C4-N0-W90, C4-N30-W90, and C4-N45-W90 specimens exhibited crushing behavior similar to that of their single-cell counterparts. In all cases, progressive failure initiated from the chamfered trigger at the top end, accompanied by corner cracking. The predominant failure mechanisms observed during the tests included fiber fracture, matrix cracking, delamination, and ply bending. Notably, the C4-N30-W90 and C4-N45-W90 specimens exhibited pronounced shear deformation within the inner sub-cells in their final crushed states.

**Figure 10:**
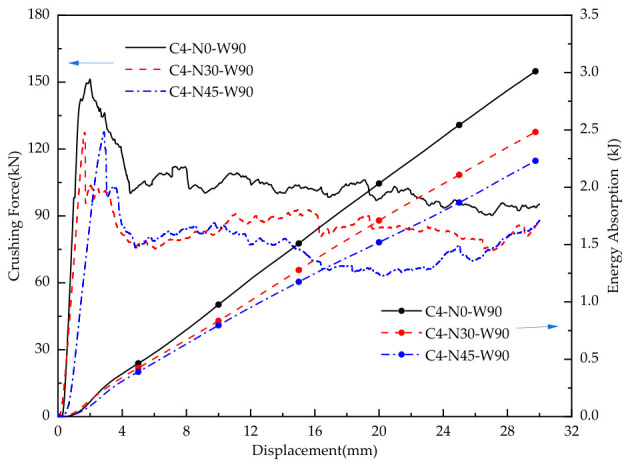
Effects of layup angle on the crushing force–displacement and *EA*–displacement responses of four-cell MAMCS.

**Figure 11:**
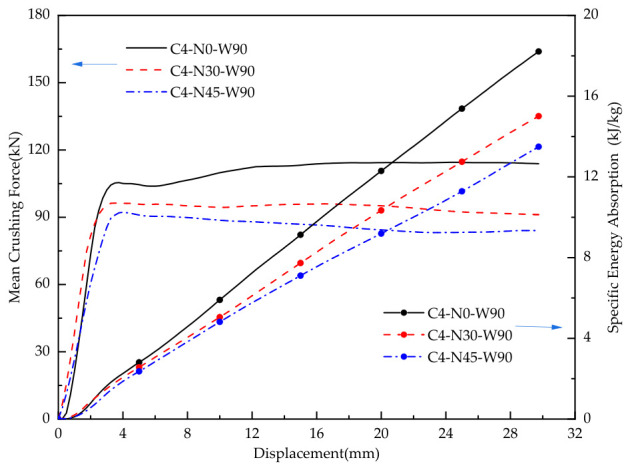
Effects of layup angle on the *MCF*–displacement and *SEA*–displacement responses of four-cell MAMCS.

The force–displacement curves of the four-cell MAMCS specimens, presented in Figure 10 and Figure 11, followed consistent trends across the three inner layup configurations. In all cases, the response mirrored that of the single-cell specimens: a sharp force drop after the initial peak, followed by a stable crushing plateau. Notably, C4-N0-W90 specimens sustained higher loads during the stable stage compared with the C4-N30-W90 and C4-N45-W90 specimens.

Quantitatively, the *MCF* of C4-N0-W90 is 24.9% and 35.6% higher than those of C4-N30-W90 and C4-N45-W90, respectively. Similarly, the *EA* and *SEA* of C4-N0-W90 are 22.0% and 35.6% higher than those of C4-N30-W90 and C4-N45-W90, respectively. These improvements were more pronounced than those observed in the single-cell specimens, primarily because the [0°/90°] layup suppresses shear deformation within the inner sub-cells, enabling more stable and efficient progressive crushing.

#### 3.3.2. Effects of Multi-Cell Configuration on Crashworthiness

**Figure 12:**
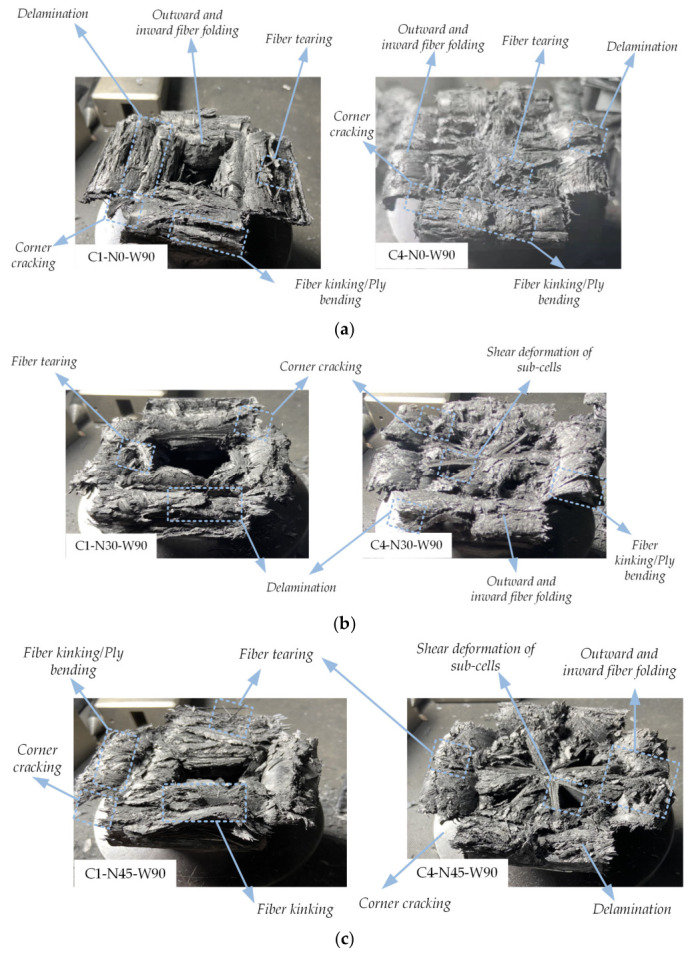
Effects of multi-cell configuration on crushing failure modes of MAMCS: (**a**) C1-N0-W90 vs. C4-N0-W90; (**b**) C1-N30-W90 vs. C4-N30-W90; (**c**) C1-N45-W90 vs. C4-N45-W90.

Figure 12a compares the deformation and failure modes of the C1-N0-W90 and C4-N0-W90 specimens. Both configurations primarily fail through similar mechanisms, including delamination, fiber tearing, corner cracking, fiber kinking, ply bending, and outward and inward fiber folding. In the four-cell configuration, the internal sub-cells are arranged in pairs, producing significant interaction effects under axial compression. These interactions promote a more stable progressive crushing process and substantially enhance the axial load-bearing capacity.

Figure 12b,c shows that the inner sub-cells in the C4-N30-W90 and C4-N45-W90 specimens undergo pronounced shear deformation. This leads to localized debonding and cracking within the inner sub-cells, as well as inward bending of the thin-walled sub-cells. Nevertheless, the multi-cell architecture allows the four internal sub-cells to mutually constrain and interact. Combined with the stabilizing effect of the outer layer, this interaction maintains circumferential stability, ensuring a stable overall progressive crushing response.

**Figure 13:**
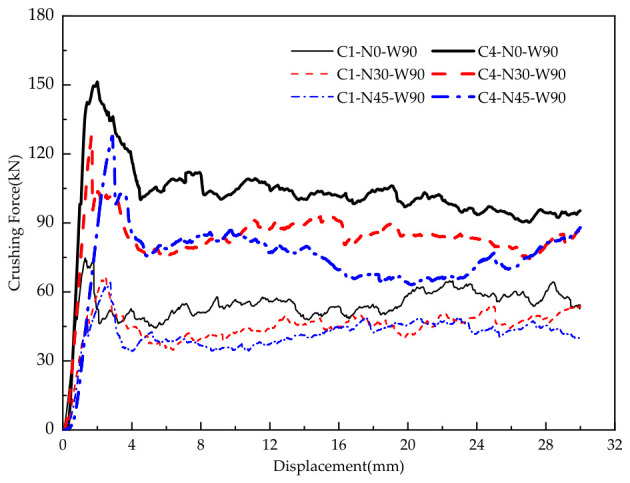
Effects of multi-cell configuration on the crushing force–displacement of MAMCS.

The force–displacement curves presented in Figure 13 reveal that the single-cell and four-cell MAMCS specimens follow similar trends. In all cases, the crushing force drops sharply after reaching the peak, followed by a stable crushing stage. However, the four-cell specimens exhibit higher crushing forces than the single-cell specimens across all three inner layup configurations, reflecting superior stability and energy absorption performance relative to their single-cell counterparts.

Quantitative comparison of the crashworthiness indicators listed in Table 2 further confirms this observation. The *MCF* and *SEA* of the four-cell specimens are substantially higher than those of the corresponding single-cell specimens. As summarized in Table 2, the *MCF* improvements for C4-N0-W90, C4-N30-W90, and C4-N45-W90 over their single-cell counterparts are 93.1%, 74.4%, and 65.7%, respectively, while the corresponding *SEA* improvements are 20.4%, 18.1%, and 16.4%. These results indicate that the four-cell MAMCS specimens exhibit superior axial load-bearing capacity and enhanced energy absorption compared with the single-cell counterparts. It is noted that the four-cell configurations were each tested once, and the reported percentage improvements should therefore be interpreted as indicative trends rather than statistically validated differences. Nevertheless, the consistently large magnitude of the improvements across all three layup configurations, the physically consistent deformation mode observations, and the independent corroboration from the validated finite element model collectively support the robustness of the reported multi-cell interaction effects.

## 4. Numerical Simulation

The experimental investigation discussed above revealed the influence of different design parameters on the axial energy absorption behavior of the modular assembled multi-cell CFRP structure (MAMCS). However, experimental methods are time-consuming and costly, which limits their efficiency for extensive parametric studies. Therefore, a numerical model of the MAMCS was developed to enhance research efficiency. The numerical model was subsequently used to investigate the effects of design parameters and to perform structural optimization.

### 4.1. Finite Element Modeling and Validation

#### 4.1.1. Finite Element Model Development

**Figure 14:**
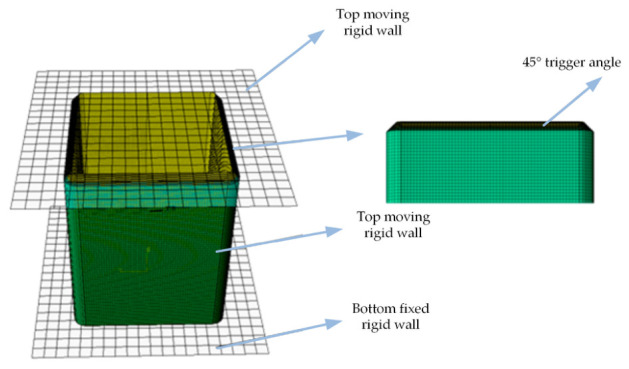
Schematic of the finite element model and boundary conditions of MAMCS.

Based on the specimen geometry and layup parameters shown in Figure 1 and Table 1, a finite element model of the MAMCS under axial crushing was developed in ABAQUS. Since the MAMCS was manufactured by stacking multiple CFRP plies, a mesoscopic modeling strategy was adopted to accurately represent the actual structural configuration. Specifically, the wall thickness was discretized into individual plies, with each ply modeled using continuum shell elements (SC8R). Cohesive elements were inserted between adjacent plies to simulate interlaminar behavior and delamination. In the four-cell configurations, cohesive elements were additionally introduced at the bond interfaces between adjacent sub-cells to simulate the mechanical behavior of the 3M DP460 epoxy adhesive layer. After a mesh convergence study, the mesh size of the MAMCS was set to 1 × 1 mm, while the loading rigid wall and fixed rigid base were meshed with an element size of 5 × 5 mm. The constitutive behavior of the CFRP plies was described by the built-in ABQ_PLY_FABRIC material model [28,29], and the evolution of interlaminar damage was characterized using a bilinear traction–separation law with mixed-mode damage governed by the Benzeggagh–Kenane (B–K) criterion. The material constants required by the ABQ_PLY_FABRIC model—including elastic moduli, strength parameters, and fracture energies—and the cohesive zone model parameters for both interlaminar and sub-cell bond interfaces were provided by Shandong Zhuoliou Carbon Fiber Products Co., Ltd., Jinan, China. based on standardized mechanical characterization of the T300/E51 prepreg system. The appropriateness of these parameters is supported by the close agreement between numerical predictions and experimental results reported in Section 4.1.2. The complete set of material parameters is not tabulated here for brevity; interested readers are encouraged to contact the corresponding author for further details. In addition, a 45° chamfered trigger was introduced at the top end of the specimen to initiate progressive crushing. The schematic of the finite element model is shown in Figure 14.

#### 4.1.2. Validation of the Numerical Model

**Figure 15:**
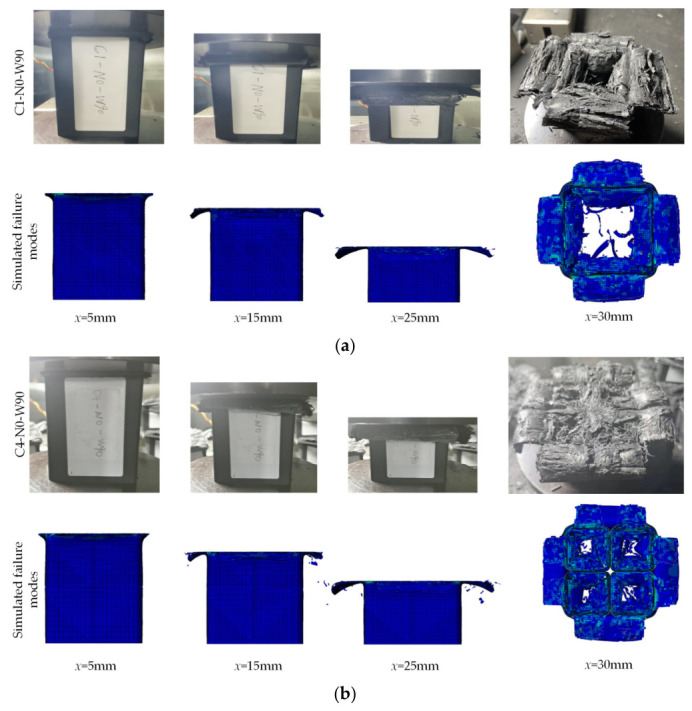
Comparison of simulated and experimental crushing deformation modes of MAMCS: (**a**) C1-N0-W90; (**b**) C4-N0-W90.

Figure 15 compares the deformation and failure modes between experimental and numerical results for the C1-N0-W90 and C4-N0-W90 specimens. The finite element model reproduces the major failure features observed in the experiments. In both the experiments and simulations, failure initiates from the chamfered trigger and propagates progressively, accompanied by inward and outward folding as well as pronounced delamination. Overall, both the experimental and numerical results exhibit a characteristic progressive petal-like crushing pattern.

**Figure 16:**
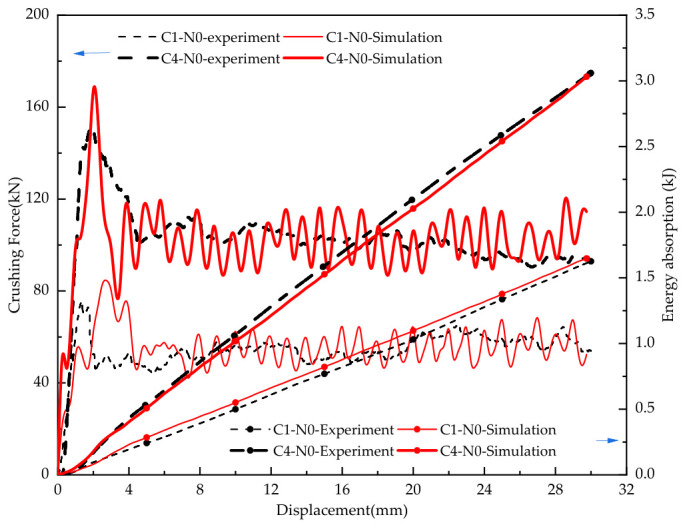
Comparison of simulated and experimental crushing force–displacement and energy absorption–displacement curves of MAMCS.

The force–displacement and energy absorption–displacement curves shown in Figure 16 further demonstrate the predictive capability of the numerical model. The differences between the numerical predictions and experimental results are conservatively within 5% for the mean crushing force (*MCF*) and total energy absorption (*EA*), and within 10% for the peak crushing force (*PCF*). It is noted that model validation is performed for the single-cell and four-cell configurations, representing the two structural extremes of the parametric study. Since all configurations share identical geometric dimensions, element formulation, and constitutive modeling framework, the predictive capability demonstrated at these two extremes is considered sufficient to support the reliability of the numerical model across the full range of configurations investigated. The repeatability of the experimental results was confirmed through duplicate tests on the C1-N0-W90 configuration (Figure 7), which showed negligible scatter between specimens, indicating that the reported 5–10% deviations between numerical and experimental results reflect modeling idealizations rather than experimental variability. This approach of validating against representative configurations and extending predictions via parametric simulation is consistent with established practice in the crashworthiness literature [13,20,22].

### 4.2. Numerical Results and Discussion

#### 4.2.1. Effects of Design Parameters on Crashworthiness

For the MAMCS shown in Figure 1, different structural configurations can be obtained by varying the dimensions of the inner thin-walled sub-cells. Because these configurations differ in their energy absorption characteristics, the cell-size parameter can be adjusted to meet different crashworthiness requirements.

As shown in Figure 1b, the configurations obtained when *a* increases from 0 to 0.5*L* are symmetric to those obtained when *a* further increases from 0.5*L* to *L*. Therefore, only the range 0 ≤ *a* ≤ 0.5*L* is considered in this study, with *a* + *b* = *L*. To investigate the effect of this structural parameter, *a* was taken as the only variable, while all other geometric dimensions and design parameters were kept constant. Five representative configurations were selected with *a* = 0, *a* = 0.125*L*, *a* = 0.25*L*, *a* = 0.375*L*, and *a* = 0.5*L*, and their corresponding geometric parameters are listed in Table 3.

**Figure 17:**
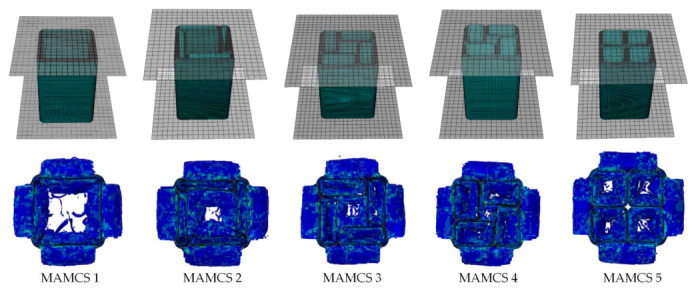
Crushing deformation modes of MAMCS specimens with different configurations.

**Figure 18:**
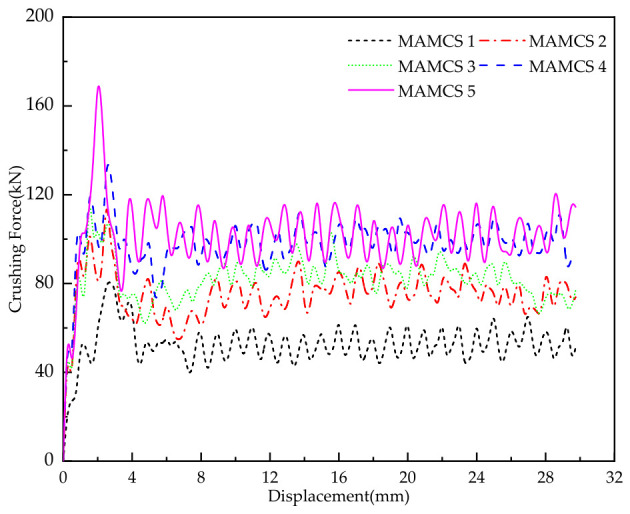
Crushing force–displacement curves of MAMCS specimens with different configurations obtained from numerical simulations.

Using the validated numerical modeling approach described above, finite element models of the five configurations listed in Table 3 were developed and analyzed. The numerical results presented in Figure 17 and Figure 18 indicate that all configurations exhibit similar failure modes, including delamination, fiber tearing, corner cracking, fiber kinking, ply bending, and outward and inward fiber folding. Owing to the adhesive bonding between the internal sub-cells, interaction effects are generated under axial compression, which promotes a more stable progressive crushing process. Detailed examination of the inner sub-cells reveals no obvious shear deformation. The outer [90°] layup is designed to maintain overall structural stability, and the simulations show pronounced circumferential cracking at the corners of the outer layer during crushing.

The force–displacement curves in Figure 18 exhibit similar overall trends for all configurations. In each case, an initial peak is followed by a sharp load drop and a relatively stable crushing plateau. Notably, the mean crushing force (*MCF*) increases progressively with increasing *a*, indicating that larger inner sub-cells improve both load-bearing capacity and energy absorption.

The key crashworthiness indicators extracted from the numerical simulations are summarized in Table 4. As shown in the table, the *PCF* increases with increasing *a*. The minimum *PCF* occurs at *a* = 0 mm (72.7 kN), while the maximum *PCF* is observed at *a* = 30 mm (159.3 kN). Since a higher *PCF* corresponds to a larger initial impact load, a lower *PCF* is generally preferred in crashworthy design. In contrast, both the *MCF* and *SEA* increase with increasing *a*, with the maximum values of 113.9 kN and 18.3 kJ/kg, respectively, both obtained at *a* = 30 mm. Notably, although *PCF*, *MCF*, and *SEA* all exhibit monotonically increasing trends with increasing *a*, these relationships are nonlinear in nature—the incremental gains in each indicator are not constant across the five configurations, reflecting a nonlinear positive correlation between *a* and the crashworthiness indicators. These results indicate that a larger *a* is beneficial for improving energy absorption, whereas a smaller *a* is preferable for reducing the peak load. Therefore, a multi-criteria approach is needed to identify the optimal configuration that balances these competing objectives. It should be noted that the parametric study for MAMCS 2–4 relies on numerical simulation, with experimental validation performed at the two geometric extremes of the parametric range (MAMCS 1 and MAMCS 5). This approach is consistent with established practice in the crashworthiness literature [13,20,22], where validated FE models are routinely employed for systematic parametric investigations. The smooth and monotonically consistent trends observed across all five configurations provide additional confidence in the reliability of the numerical predictions for the intermediate cases.

#### 4.2.2. Optimal Parameter Selection Using MCDM Method

The crashworthiness of the MAMCS is characterized by multiple indicators, including the *PCF*, *MCF*, and *SEA*. Therefore, the optimal structural configuration cannot be determined based on a single indicator. In practical crashworthy design, *PCF* should be minimized to reduce the initial impact load, whereas *MCF* and *SEA* should be maximized to improve the load-bearing capacity and energy absorption efficiency. Accordingly, a multi-criteria decision-making (MCDM) method was employed to determine the preferred configuration.

Specifically, the Technique for Order Preference by Similarity to the Ideal Solution (TOPSIS) was adopted to identify the optimal configuration. TOPSIS is widely used in engineering optimization because of its simplicity, computational efficiency, and ability to rank alternatives according to their relative distances from the positive and negative ideal solutions [30,31]. In this study, the evaluation criteria include the *PCF*, *MCF*, and *SEA*. *PCF* was treated as a cost-type criterion (to be minimized), whereas *MCF* and *SEA* were treated as benefit-type criteria (to be maximized).

**Step 1: Construction of the decision matrix.**

The decision matrix X was constructed from the crashworthiness indicators summarized in Table 4:

(4) $$X=\left[x_{ij}\right]$$

where *x_ij_* represents the value of the *j*-th indicator for the *i*-th configuration.

**Step 2: Normalization of the decision matrix.**

To eliminate the dimensional differences among the indicators, vector normalization was applied to each column of the decision matrix:

(5) rij=xij∑i=05xij2

where *r_ij_* is the normalized value listed in Table 5.

**Step 3. Construction of the weighted normalized matrix.**

Considering that *SEA* is the most representative indicator of energy absorption efficiency for lightweight structures, the weights assigned to *PCF*, *MCF*, and *SEA* were 0.25, 0.25, and 0.5, respectively. Therefore, the weight vector was defined as *w*_1_ = [0.25, 0.25, 0.5]. To assess the robustness of the ranking, two additional weighting scenarios were considered: *w*_2_ = [0.2, 0.2, 0.6], which places greater emphasis on *SEA*, and *w*_3_ = [0.3, 0.3, 0.4], which assigns higher importance to *PCF* and *MCF*. The weighted normalized matrix was then calculated as *v_ij_* = *w*⋅*r_ij_*.

**Step 4. Determination of the ideal solutions.**

Based on the weighted normalized matrix, the positive ideal solution *A*^+^ and the negative ideal solution *A*^−^ were determined [30].

**Step 5. Calculation of the relative closeness.**

The separation distances from the positive and negative ideal solutions were calculated, and the relative closeness *C_i_* was obtained as follows:

(6) Ci=Si−(Si++Si−)

where *S_i_*^+^ and *S_i_*^−^ denote the Euclidean distances from the *i*-th alternative to the positive and negative ideal solutions, respectively. The calculated TOPSIS results are summarized in Table 5.

As shown in Table 5, under the baseline weighting scenario *w*_1_ = [0.25, 0.25, 0.5], MAMCS 4 achieves the highest relative closeness coefficient, followed by MAMCS 5, MAMCS 1, MAMCS 3, and MAMCS 2. Although MAMCS 5 exhibits the highest *MCF* and *SEA* values, it also produces the largest *PCF*, which penalizes its overall ranking due to the cost-type nature of this criterion. By contrast, MAMCS 4 provides a better balance between peak load reduction and energy absorption capability.

To further assess the robustness of this ranking, the TOPSIS analysis was repeated under two additional weighting scenarios, and the resulting relative closeness coefficients are summarized in Table 5. Under *w*_2_ = [0.2, 0.2, 0.6], which places greater emphasis on *SEA*, MAMCS 5 marginally outperforms MAMCS 4; however, this comes at the cost of a substantially higher *PCF* (159.3 kN vs. 130.9 kN), which is generally undesirable in crashworthy design. Under *w*_3_ = [0.3, 0.3, 0.4], which assigns higher importance to *PCF* and *MCF*, MAMCS 1 becomes preferred due to its lowest *PCF*, while MAMCS 4 remains second. Across all three weighting scenarios, MAMCS 4 consistently ranks among the top two configurations, demonstrating the robustness of this selection. For automotive and aerospace crashworthy applications where both energy absorption efficiency and peak load mitigation are critical design objectives, the baseline weighting *w*_1_ represents a balanced engineering judgment, under which MAMCS 4 is identified as the preferred configuration. It should be noted that this recommendation is conditional on the adopted weighting scheme, and alternative weighting priorities may lead to different ranking outcomes, as demonstrated by the sensitivity analysis summarized in Table 5.

## 5. Conclusions

In this study, a novel modular assembled multi-cell CFRP structure (MAMCS) inspired by the topology of natural honeycombs was developed. Quasi-static axial crushing experiments and numerical simulations were conducted to evaluate its crashworthiness. The effects of inner layup configurations, cell number, and structural evolution parameters on crushing performance were systematically investigated. The main conclusions are summarized as follows:

(1) The inner layup configuration and cell number significantly influence the crashworthiness of the MAMCS. Among the investigated configurations, the [0°/90°] inner layup exhibited the best overall performance, with higher *MCF* and *SEA* than the [30°/−60°] and [45°/−45°] layups. In addition, the modular four-cell structure demonstrated superior load-bearing capacity and energy absorption efficiency compared with the single-cell configuration, mainly due to the stabilizing interaction effects among the internal sub-cells.
(2) A finite element model was developed and validated against experimental results for two representative configurations (C1-N0-W90 and C4-N0-W90), representing the single-cell and four-cell extremes of the parametric study. The simulations successfully captured the dominant deformation and failure modes observed in the tests, and the predicted force–displacement responses were in close agreement with the experimental data, with deviations within 5% for *MCF* and *EA* and within 10% for *PCF*.
(3) Parametric studies revealed that the inner sub-cell size parameter *a* has a pronounced influence on crashworthiness. Increasing *a* leads to higher *MCF* and *SEA* values, while also increasing the *PCF*, reflecting a nonlinear positive correlation and an inherent trade-off between energy absorption efficiency and peak load mitigation. Using the TOPSIS-based multi-criteria decision-making framework with a baseline weighting that prioritizes *SEA* while equally balancing *PCF* and *MCF*, the configuration MAMCS 4 was identified as the preferred configuration under the specified engineering priorities, providing a favorable balance between peak load reduction and energy absorption capability.

The proposed MAMCS demonstrates excellent crashworthiness with high energy absorption efficiency and structural stability, enabled by a simple and cost-effective modular assembly strategy. The present work offers a promising bio-inspired design concept for lightweight energy-absorbing structures in automotive and aerospace applications. It should be noted that the present study has two principal limitations: the experimental campaign involved a limited number of replications per configuration, and the investigation was conducted under quasi-static loading conditions only, which may not fully represent the structural response under dynamic impact scenarios. Future work will focus on dynamic impact loading, further structural optimization, the systematic investigation of a broader range of multi-cell configurations with varying cell numbers and arrangements, and a more comprehensive experimental validation campaign.

## Figures and Tables

**Table 1:** Layup configurations of the MAMCS specimens.

Specimen ID	Inner Sub-Cells Layup	Outer Wrapping Layup	Ply Thickness (mm)
C1-N0-W90	[0°/90°]_5_	[90°]_6_	0.2
C1-N30-W90	[30°/−60°]_5_	[90°]_6_	0.2
C1-N45-W90	[45°/−45°]_5_	[90°]_6_	0.2
C4-N0-W90	[0°/90°]_5_	[90°]_6_	0.2
C4-N30-W90	[30°/−60°]_5_	[90°]_6_	0.2
C4-N45-W90	[45°/−45°]_5_	[90°]_6_	0.2

**Table 2:** Crashworthiness indicators of all MAMCS specimens.

Specimen ID	*PCF* (kN)	*MCF* (kN)	*SEA* (kJ/kg)
C1-N0-W90	72.7	59.0	15.2
C1-N30-W90	65.9	52.3	12.7
C1-N45-W90	64.0	50.7	11.6
C4-N0-W90	159.3	113.9	18.3
C4-N30-W90	127.0	91.2	15.0
C4-N45-W90	127.6	84.0	13.5

**Table 3:** MAMCS configurations for different design parameters.

Configuration ID	*a* (mm)	*b* (mm)	Axial Length *L* (mm)	Inner and Outer Layer Thickness (mm)
MAMCS 1	0	60	60	2/1.2
MAMCS 2	7.5	52.5	60	2/1.2
MAMCS 3	15	45	60	2/1.2
MAMCS 4	22.5	37.5	60	2/1.2
MAMCS 5	30	30	60	2/1.2

**Table 4:** Crashworthiness indicators of MAMCS with different configurations.

Configuration ID	*PCF* (kN)	*MCF* (kN)	*SEA* (kJ/kg)
MAMCS 1	72.7	59.0	15.2
MAMCS 2	116.3	75.5	15.3
MAMCS 3	119.0	83.5	16.2
MAMCS 4	130.9	96.4	17.4
MAMCS 5	159.3	113.9	18.3

**Table 5:** TOPSIS evaluation results and ranking of the MAMCS configurations (Bold indicates the top two configurations across all weighting scenarios).

Configuration ID	*r*_PCF_	*r*_MCF_	*r*_SEA_	*w*_1_ *C_i_*/Rank	*w*_2_ *C_i_*/Rank	*w*_3_ *C_i_*/Rank
MAMCS 1	0.2646	0.3010	0.4114	0.4912/3	0.4555/3	**0.5111/1**
MAMCS 2	0.4233	0.3852	0.4141	0.3722/5	0.3368/5	0.3930/5
MAMCS 3	0.4331	0.4260	0.4384	0.4391/4	0.4222/4	0.4485/4
MAMCS 4	0.4764	0.4918	0.4709	**0.5130/1**	**0.5378/2**	**0.4989/2**
MAMCS 5	0.5798	0.5811	0.4953	**0.5008/2**	**0.5446/1**	0.4889/3

## Data Availability

The original contributions presented in this study are included in the article. Further inquiries can be directed to the corresponding author.
